# Impact of an eHealth Smartphone App on Quality of Life and Clinical Outcome of Patients With Hand and Foot Eczema: Prospective Randomized Controlled Intervention Study

**DOI:** 10.2196/38506

**Published:** 2023-03-07

**Authors:** Wanja Alexander Weigandt, Yannic Schardt, Aimee Bruch, Raphael Herr, Matthias Goebeler, Johannes Benecke, Astrid Schmieder

**Affiliations:** 1 Department of Dermatology, University Medical Center Mannheim, Heidelberg University Mannheim Germany; 2 Center for Preventive Medicine and Digital Health Baden-Württemberg (CPD-BW), Medical Faculty Mannheim, Heidelberg University Mannheim Germany; 3 Department of Dermatology Venereology and Allergology University Hospital Würzburg Würzburg Germany

**Keywords:** hand and foot eczema, eHealth, mobile health, mHealth, telemedicine, disease management, smartphone app, mobile phone

## Abstract

**Background:**

Chronic hand and foot eczema is a polyetiological dermatological condition. Patients experience pain, itching, and sleep disturbances and have a reduced quality of life. Skin care programs and patient education can improve the clinical outcome. eHealth devices offer a new opportunity to better inform and monitor patients.

**Objective:**

This study aimed to systematically analyze the effect of a monitoring smartphone app combined with patient education on the quality of life and clinical outcome of patients with hand and foot eczema.

**Methods:**

Patients in the intervention group received an educational program; attended study visits on weeks 0, 12, and 24; and had access to the study app. Patients in the control group attended the study visits only. The primary end point was a statistically significant reduction in Dermatology Life Quality Index, pruritus, and pain at weeks 12 and 24. The secondary end point was a statistically significant reduction in the modified Hand Eczema Severity Index (HECSI) score at weeks 12 and 24. This is an interim analysis at week 24 of the 60-week randomized controlled study.

**Results:**

In total, 87 patients were included in the study and randomized to the intervention group (n=43, 49%) or control group (n=44, 51%). Of the 87 patients, 59 (68%) completed the study visit at week 24. There were no significant differences between the intervention and control groups regarding quality of life, pain, itch, activity, and clinical outcome at weeks 12 and 24. Subgroup analysis revealed that, compared with the control group, the intervention group with an app use frequency of fewer than once every 5 weeks had a significant improvement in the Dermatology Life Quality Index at weeks 12 (*P*=.001) and 24 (*P*=.05), in pain measured on a numeric rating scale at weeks 12 (*P*=.02) and 24 (*P*=.02), and in the HECSI score at week 12 (*P*=.02). In addition, the HECSI scores assessed on the basis of pictures taken by the patients of their hands and feet correlated strongly with the HECSI scores recorded by physicians during regular personal visits (*r*=0.898; *P*=.002) even when the quality of the images was not that good.

**Conclusions:**

An educational program combined with a monitoring app that connects patients with their treating dermatologists can improve quality of life if the app is not used too frequently. In addition, telemedical care can at least partially replace personal care in patients with hand and foot eczema because the analysis of the pictures taken by the patients correlates strongly with that of the in vivo images. A monitoring app such as the one presented in this study has the potential to improve patient care and should be implemented in daily practice.

**Trial Registration:**

Deutsches Register Klinischer Studien DRKS00020963; https://drks.de/search/de/trial/DRKS00020963

## Introduction

### Background

The prevalence of combined chronic hand and foot eczema in industrialized cities is 5.4% [1]. Women are more frequently affected than men, with an incidence of 9.6 per 1000 compared with 4.0 per 1000 [2].

Hand and foot eczema is considered to be chronic if it persists for >3 months despite adequate therapy or recurs with a frequency of more than twice a year [3]. It does not represent a homogeneous disease entity. The clinical picture, morphology, localization, and etiology can be very different. In general, 4 different etiologies of hand and foot eczema exist: allergic contact, acute-toxic, cumulative-toxic, and atopic hand and foot eczema [4]. Allergic contact hand and foot eczema is typically a type IV sensitization to diverse allergens such as nickel, cobalt, chromates, and fragrancies [5]. Cumulative-toxic hand and foot eczema occurs after repeated exposure to substances that only mildly irritate the skin. Over time, the regenerative capacity of the skin is exceeded, and the eczematous reaction becomes visible. Atopic hand and foot eczema develops on the basis of a genetic predisposition called atopic diathesis. It is therefore a localized variant of atopic eczema with a corresponding etiology [3,6].

The severity of eczema ranges from very mild to very severe, with therapy-refractory courses associated with intense pain and itching [7]. In addition, patients with eczema often have to face social stigmatization and struggle with feelings of shame [8]. These physical and psychological circumstances often lead to a radical reduction in quality of life and may even result in depression [9].

More often than not, patients with eczema have limited knowledge of the pathogenesis of their skin condition and the correct disease management [10]. In many other diseases such as type 2 diabetes mellitus, patient education has proven to be an effective method to increase knowledge of the disease, thereby improving the clinical outcome. Coppola et al [11] have shown that patient education is usually associated with an improvement in clinical knowledge, lifestyle, and psychosocial outcomes in comparison with usual care. In Germany, there are skin protection seminars run by employers’ liability insurance associations, but these are reserved for people whose eczema is caused or exacerbated by their professional activity.

In our department of dermatology, patient education alone for patients with psoriasis had no significant effect on the clinical outcome [12]. We therefore assume that one-time education of patients with chronic inflammatory skin conditions may not suffice to ameliorate the disease in the long term.

eHealth-based supporting systems for patients are becoming popular and are incorporated more frequently into patient care. Germany recently set up the German acronym for Digital Health Applications (DiGA) directory, which lists Conformité Européenne–marked medical devices that aim to detect, monitor, treat, or alleviate diseases or to detect, treat, alleviate, or compensate for injuries or disabilities [13]. Physicians (MDs) in Germany can prescribe eHealth devices listed in the DiGA directory. There are currently no DiGA directory–listed eHealth devices for patients experiencing hand and foot eczema in Germany, and scientific data on the beneficial effect of eHealth applications for these patients are missing.

### Objectives

The aim of this prospective randomized controlled intervention study was to analyze whether a monitoring smartphone app combined with patient education would improve the quality of life and clinical outcome of patients with hand and foot eczema. The study app was developed specifically for this study. With the app, our patients were able to periodically measure Dermatology Life Quality Index (DLQI) and Hand Eczema Severity Index (HECSI; modified version for foot eczema) scores, as well as the impact on activity and pain (both measured on a numeric rating scale [NRS]), and document the progression of their disease through photographs [14-16]. In addition, the app allowed patients to directly contact their own treating physicians through a chat function.

Furthermore, the DLQI, HECSI, and NRS (for activity and pain) scores were assessed by the treating physicians during personal visits at weeks 0 (before the intervention), 12, and 24.

The final aim behind the development of the app was to reduce waiting time for a physician’s appointment in case of an emergency by expanding teledermatological services for patients with hand and foot eczema and to allow precise self-monitoring by the patients.

## Methods

### Study Design

The aim of this 60-week randomized controlled intervention study was to investigate the effect of patient education in combination with a monitoring smartphone app on patients experiencing chronic hand and foot eczema. This is an interim analysis of the data from study weeks 0, 12, and 24.

The study was carried out at the department of dermatology, venereology, and allergology at the University Medical Center Mannheim in Mannheim, Germany, from August 13, 2018, to August 30, 2021. The inclusion criteria included a physician-confirmed diagnosis of chronic hand and foot eczema, ability to give informed consent, access to a smartphone, and patient age between 18 and 75 years. During the first study visit (week 0 [V1]), the study participants were randomly assigned to the control or intervention group in a ratio of 1:1.

To assign patients to a group, we created 50 lots for the intervention group and 50 lots for the control group. These were sealed in an urn, and the patients were asked to draw lots.

In total, 90 participants were included in the study, but 3 (3%) dropped out of the study before they were assigned to a group. Of the 87 remaining participants, 43 (49%) were assigned to the intervention group and 44 (51%) to the control group.

The control group started the first study visit at week 0. Information on sociodemographic data, preexisting conditions, and previous and current therapies were collected, and standardized questionnaires such as the DLQI administered. In addition, patients’ current level of knowledge about their disease, severity of the disease measured using the HECSI or a modified form of the HECSI for foot eczema, and the intensity of the pain and itch measured using an NRS ranging from 0 to 10 were recorded. Furthermore, the negative impact on the activity measured using the NRS of patients was assessed. In-person follow-up visits were carried out at V2 and V3. The same parameters were recorded for the intervention group. In addition, these patients received a 2-hour detailed training session on pathogenesis, classification, therapeutic options, and behavioral recommendations from 2 dermatological specialists at our clinic. Each patient also received a personal access code to our app, DermaScope Mobile. Using this app, patients were able to take pictures of their hands and feet, use a chat function to ask questions that were answered by their treating dermatologists, and complete questionnaires on quality of life (DLQI) and current symptoms (NRS for itch and pain). Screenshots of the app can be found in the paper by Domogalla et al [17]. The highest possible app use frequency was once a week.

The quality of each image uploaded in the app by the patients was categorized by the rater (YS) as good or bad based on the following criteria: well-illuminated picture, sharp and focused image, and complete presentation of the hands and feet. All 3 criteria had to be met for the image to be rated as good. Each image was assigned to the rater (YS), who checked its quality based on these 3 criteria. If all criteria were met, the image was rated as of good quality. We then calculated an electronic HECSI (eHECSI) score based on these images and statistically examined the extent to which this score correlated with the HECSI score collected in person.

The primary end point of the study was to determine the effect of extensive patient training, physician-patient contact on demand, and our app on quality of life as well as itching and pain at weeks 12 and 24. The secondary end points were the effect on the disease outcome assessed with the HECSI at weeks 12 and 24. Modulating effects of sex, age, and disease duration were evaluated for each end point.

### Ethics Approval

The medical ethics committee of the Medical Faculty Mannheim, Heidelberg University, approved the study (2017-655N-MA), and the implementation complied with the Declaration of Helsinki. All participants were instructed in detail regarding the study design and gave their informed consent before participating in the study.

### Statistical Analysis

Linear panel data regression analyses estimated the trajectories in the outcomes. Random effect regressions determined the main and interaction effects of group membership (intervention vs control group) and visit time point (V1, V2, and V3) on DLQI, pain, daily activity, and HECSI scores. Two models of adjustment were calculated. The first model was unadjusted, whereas the second model was adjusted for sex, age, and disease duration. In additional analyses, the effects of app use frequency over 24 weeks were included (group membership: control vs <20% app use frequency vs ≥20% app use frequency). Therefore, the intervention group was divided into 2 groups: one comprised patients with app use frequency <20%, and the other was made up of patients with app use frequency ≥20% during the observation period of 24 weeks. The chosen cutoff of 20% equals app use frequency of once every 5 weeks. Variables were tested for normal distribution, and where relevant, they were transformed to approach normal distribution (power transform square root of DLQI and log_10_ of HECSI). All statistical analyses were performed using STATA Special Edition (version 14.0; StataCorp LLC).

To determine the extent to which the eHECSI score correlated with the HECSI score assessed at the face-to-face visit, we calculated Spearman correlation coefficients.

We also examined within the intervention group the socioeconomic factors that influenced the course of HECSI and DLQI.

**Figure 1:**
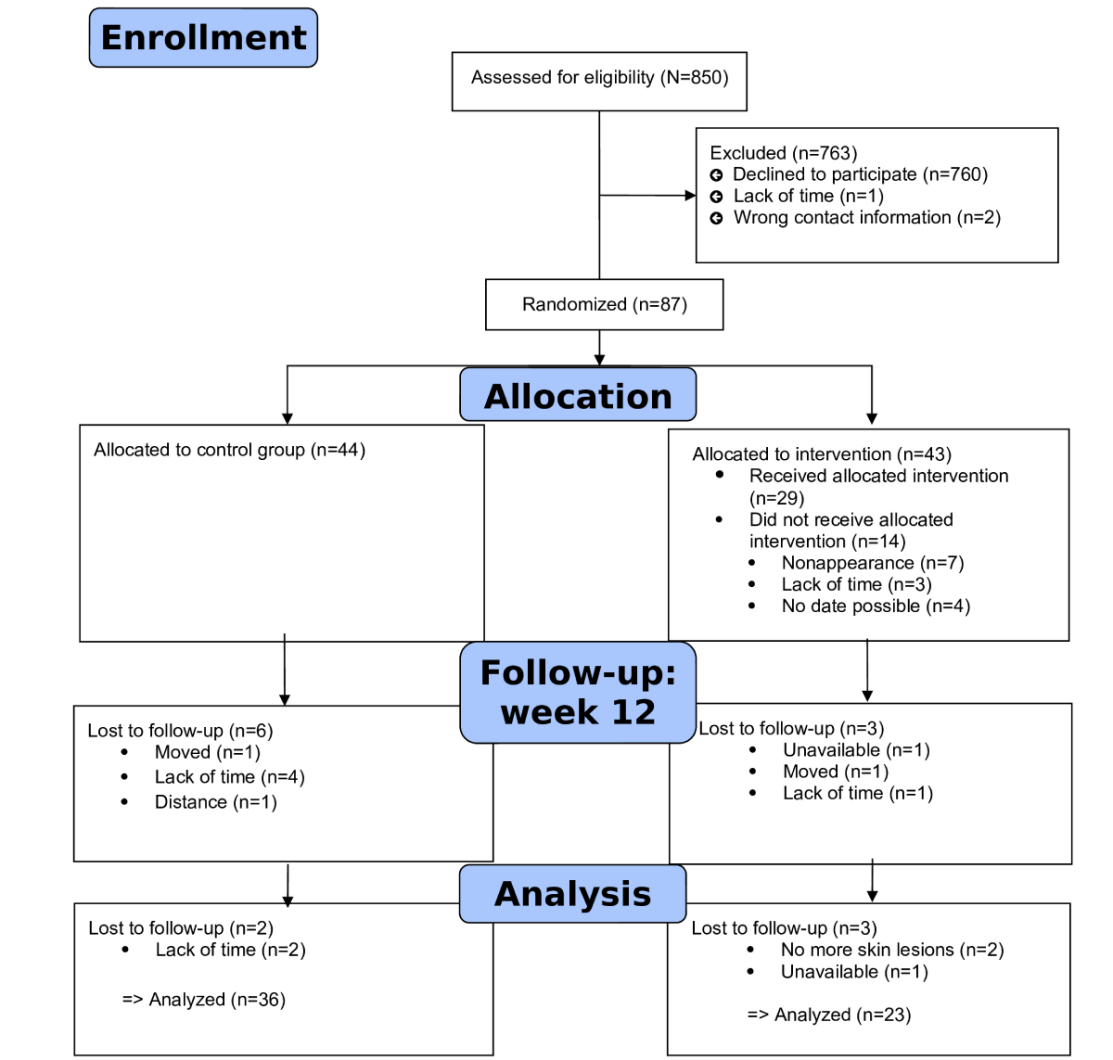
Flow chart of the study cohort and subcohorts.

**Figure 2:**
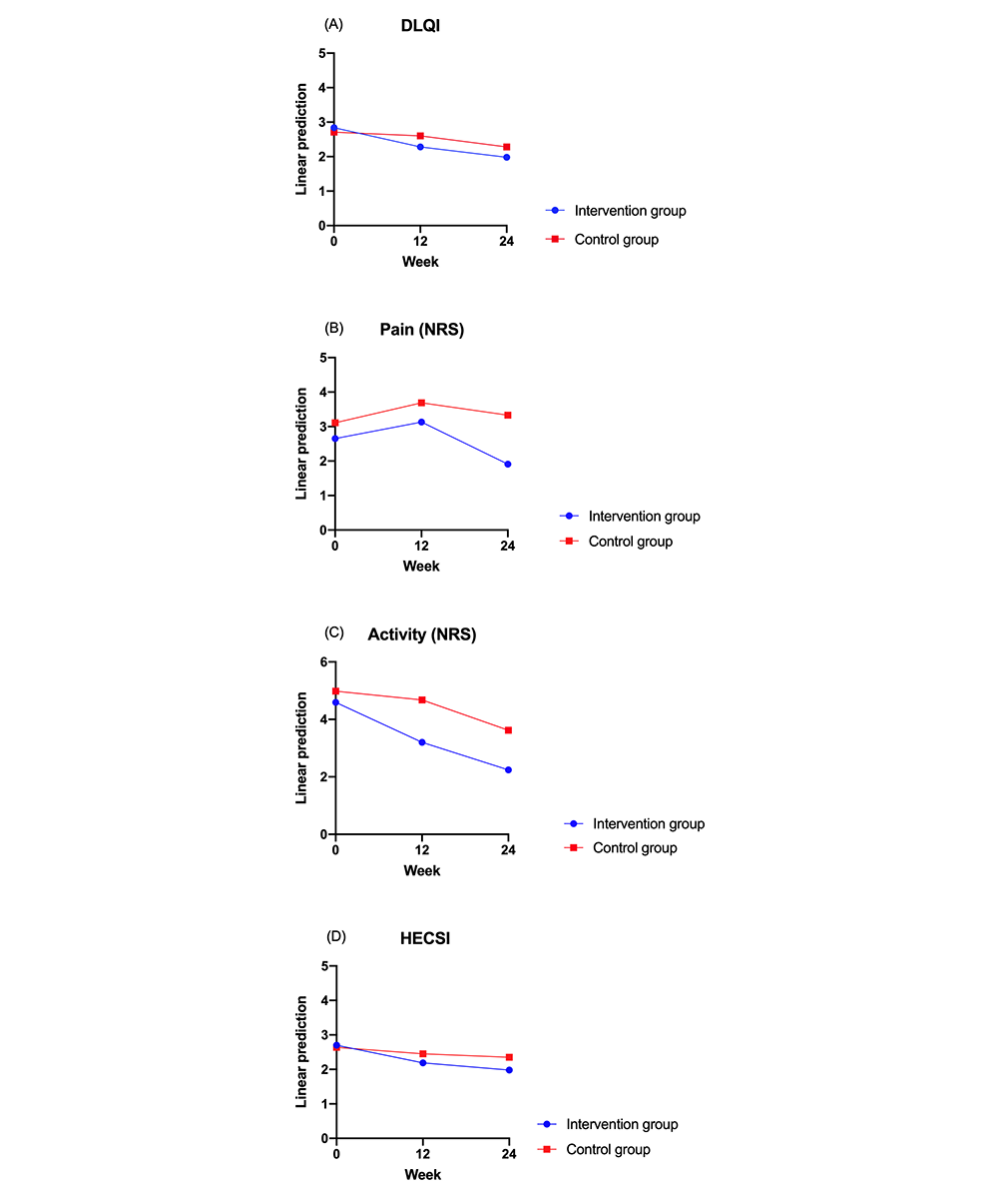
Progression of Dermatology Life Quality Index (DLQI), pain, activity, and Hand Eczema Severity Index (HECSI) in the control group (n=36) versus that in the intervention group (n=23). (A) Progression of DLQI over 24 weeks in the intervention group compared with that in the control group. Changes in both groups from baseline were significant (week 12: *P*=.006; week 24: *P*<.001). There were no significant differences between the groups (week 12: *P*=.09; week 24: *P*=.11). (B) Progression of pain scores over 24 weeks in the intervention group compared with that in the control group. Changes in both groups from baseline were not significant (week 12: *P*=.48; week 24: *P*=.28). There were no differences between the groups (week 12: *P*=.90; week 24: *P*=.27). (C) Progression of activity scores over 24 weeks in the intervention group compared with that in the control group. Changes in both groups from baseline were significant (week 12: *P*=.04; week 24: *P*=.001). There were no significant differences between the groups (week 12: *P*=.21; week 24: *P*=.26). (D) Progression of HECSI over 24 weeks in the intervention group compared with that in the control group. Changes in both groups from baseline were significant (week 12: *P*=.03; week 24: *P*=.002). There were no significant differences between the groups (week 12: *P*=.26; week 24: *P*=.14). Significance at *P*<.05. NRS: numeric rating scale.

**Figure 3:**
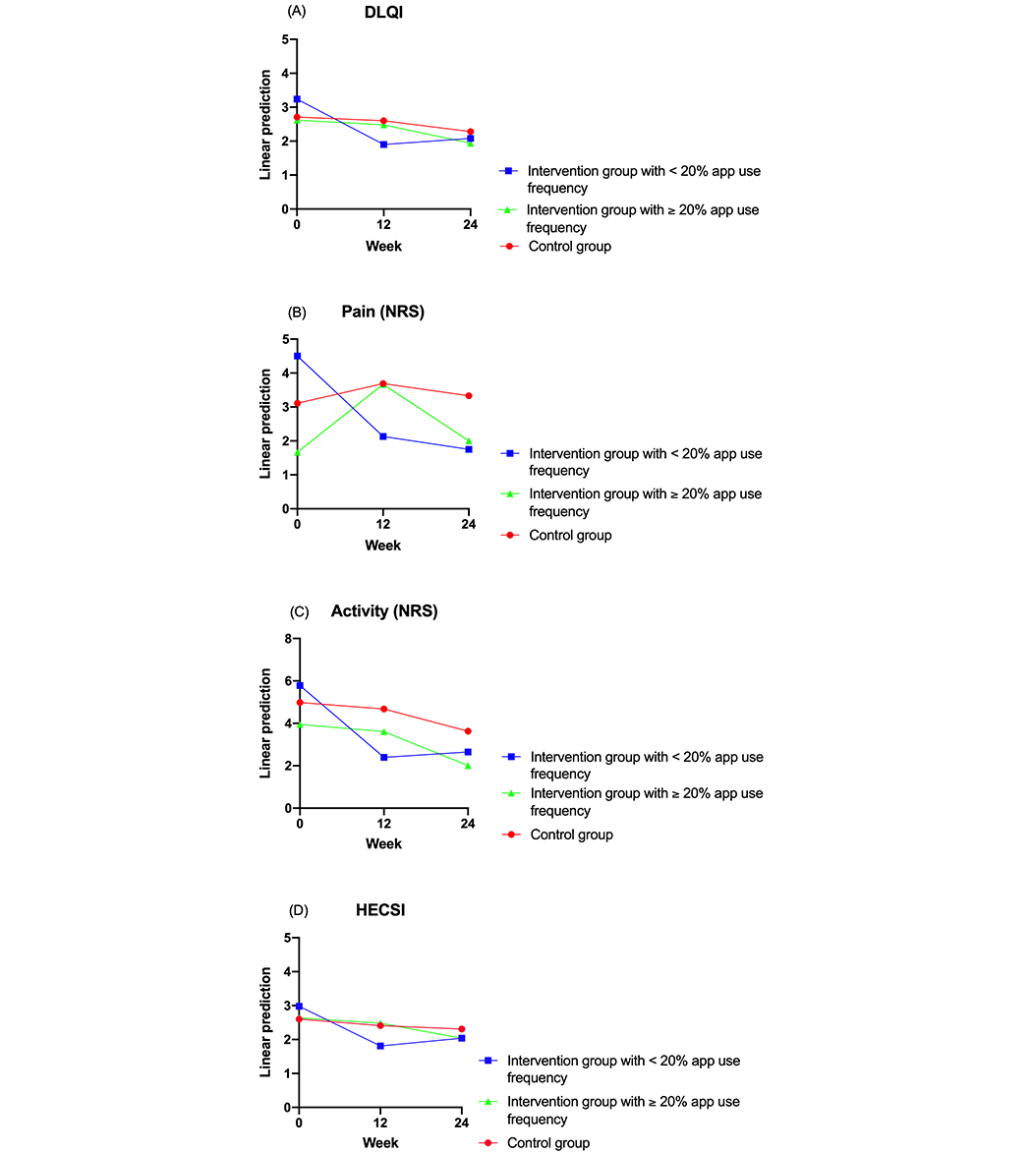
Progression of Dermatology Life Quality Index (DLQI), pain, activity, and Hand Eczema Severity Index (HECSI) in the control group (n=36) versus that in the intervention group with <20% app use frequency (n=8) versus that in the intervention group with ≥20% app use frequency (n=15). (A) Progression of DLQI over 24 weeks in the intervention group with <20% app use frequency compared with that in the intervention group with ≥20% app use frequency compared with that in the control group. Changes were significant in the <20% app use frequency group (week 12: *P*=.001; week 24: *P*=.049) but not in the ≥20% app use frequency group (week 12: *P*=.91; week 24: *P*=.39) compared with controls. (B) Development of pain scores over 24 weeks in the intervention group with <20% app use frequency compared with that in the intervention group with ≥20% app use frequency compared with that in the control group. Changes were significant in the <20% app use frequency group (week 12: *P*=.02; week 24: *P*=.02) but not in the ≥20% app use frequency group (week 12: *P*=.14; week 24: *P*=.91). (C) Development of activity scores over 24 weeks in the intervention group with <20% app use frequency compared with that in the intervention group with ≥20% app use frequency compared with that in the control group. Changes in the <20% app use frequency group were significant at week 12 but not at week 24 (week 12: *P*=.01; week 24: *P*=.17), whereas in the ≥20% app use frequency group (week 12: *P*=.98; week 24: *P*=.56), there were no significant differences. (D) Progression of HECSI over 24 weeks in the intervention group with <20% app use frequency compared with that in the intervention group with ≥20% app use frequency compared with that in the control group. Changes in the <20% app use frequency group were significant at week 12 but not at week 24 (week 12: *P*=.02; week 24: *P*=.12). There were no significant differences in the ≥20% app use frequency group (week 12: *P*=.94; week 24: *P*=.35). Significance at *P*<.05. NRS: numeric rating scale.

**Figure 4:**
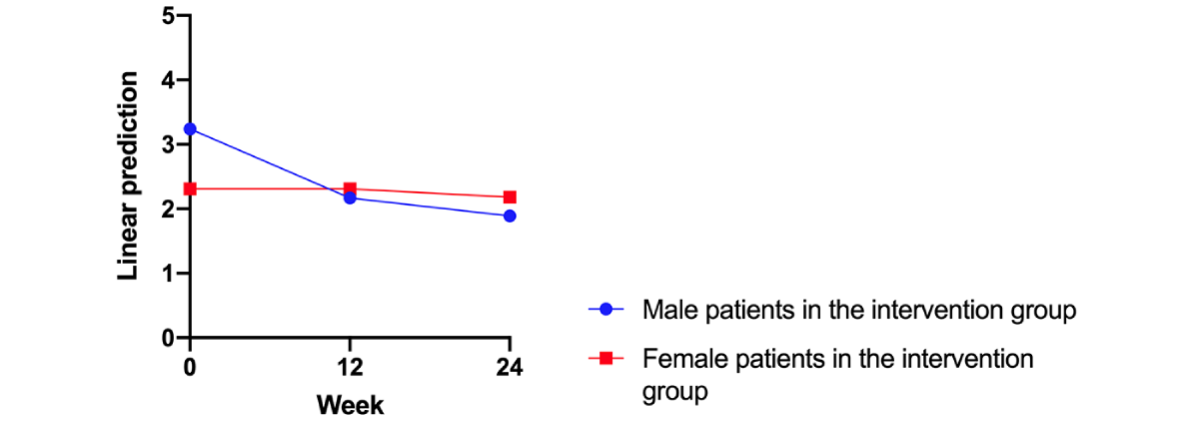
Sex-specific progression of the Hand Eczema Severity Index in the intervention group over 24 weeks. Female participants in the intervention group were compared with male participants. Changes were significant only for the male participants (week 12: *P*=.008; week 24: *P*=.003). Significance at *P*<.05.

Table 1 shows mean values of the scales, Figure 1 shows the flowchart of the study, and Figures 2-4 show predictive margins (delta method).

**Table 1:** Patient characteristics.

		Week 0 (V1^a^)				Week 24 (V3)		
		Overall (n=87)	Control group (n=44)	Intervention group (n=43)	Overall (n=59)		Control group (n=36)	Intervention group (n=23)
**Sex, n (%)**								
	Female	51 (59)	25 (57)	26 (61)	33 (56)		21 (58)	12 (52)
	Male	36 (41)	19 (43)	17 (40)	26 (44)		15 (42)	11 (48)
**Age (years)**								
	Mean (SD)	47.07 (15.42)	48.05 (14.09)	46.07 (16.78)	51.05 (13.98)		50.28 (12.84)	52.26 (15.82)
	Median	50	51	49	54		53	54
**BMI (kg/m^2^)^b^**								
	Mean (SD)	27.62 (7.53)	26.45 (5.7)	28.82 (9.2)	27.60 (8.17)		26.63 (5.70)	29.11 (10.97)
	Median	25.78	25.16	26.81	25.82		25.3	26.81
Smoker, n (%)^b^		29 (33)	16 (36)	13 (30)	20 (34)		13 (37)	7 (30)
**Duration of eczema (years)^b^**								
	Mean (SD)	6.9 (8.23)	6.0 (8.47)	7.81 (7.98)	8.02 (9.27)		6.67 (9.09)	10.13 (9.34)
	Median	4	3	6	4		4	8
**Antieczema therapy, n (%)**								
	Topical urea	78 (90)	41 (93)	37 (86)	56 (95)		35 (97)	21 (91)
	Topical glucocorticoids	57 (66)	30 (68)	27 (63)	41 (70)		26 (72)	15 (62)
	Topical calcineurin inhibitor	14 (16)	9 (21)	5 (12)	12 (20)		8 (22)	4 (17)
	Systemic therapy	10 (12)	3 (7)	7 (16)	7 (12)		3 (8)	4 (17)
**DLQI^c^ (scores range from 0 to 30)**								
	Mean (SD)	7.97 (6.38)	7.73 (7.16)	8.21 (5.55)	4.71 (5.38)		5 (5.39)	4.26 (5.5)
	Median	6	6	8	3		3.5	2
**Pain (scores range from range 0 to 10)**								
	Mean (SD)	1.94 (2.67)	2.14 (2.77)	1.74 (2.59)	1.78 (2.61)		2.33 (2.97)	0.91 (1.65)
	Median	0	1	0	0		1	0
**Activity (scores range from range 0 to 10)**								
	Mean (SD)	4.02 (3.23)	3.95 (3.37)	4.09 (3.12)	2.08 (2.60)		2.50 (2.90)	1.43 (1.93)
	Median	4	4	4	1		1.5	1
**HECSI^d^ (scores range from0 to 360)**								
	Mean (SD)	22.53 (21.29)	20.93 (20.72)	24.16 (21.99)	14.58 (18.44)		17.00 (21.67)	10.80 (11.16)
	Median	18	15	19	8		9	6
**App use frequency^e^, n (%)**								
	<20%	N/A^f^	N/A	N/A	8 (14)		0 (0)	8 (35)
	≥20%	N/A	N/A	N/A	15 (25)		0 (0)	15 (65)

^a^V: visit time point.

^b^Data for BMI, smoking, and eczema duration were collected at the first visit only.

^c^DLQI: Dermatology Life Quality Index.

^d^HECSI: Hand Eczema Severity Index.

^e^Data for app use frequency were calculated over the whole 24 weeks.

^f^N/A: not applicable.

## Results

### Patient Demographics

In total, 90 patients were included in the study. The main reasons for declining participation were lack of time, amelioration of hand and foot eczema, or distance to our outpatient clinic.

Of the 90 patients who signed the informed consent form, 87 (97%) took part in the baseline visit and were randomized 1:1 to the intervention (n=43, 49%) or control (n=44, 51%) groups. Of the 90 patients initially included in the study, 3 (3%) dropped out of the study before the baseline visit. Of the 87 remaining patients, 23 (26%) discontinued the study after the baseline visit or the educational program (intervention group: 17/43, 40%, and control group: 6/44, 14%). Leading up to week 24, of the 64 remaining patients, 5 (8%) discontinued the study, resulting in 59 (92%) patients completing the week 24 visit (Figure 1).

### Effects of the Intervention on Quality of Life, Pain, Activity, and Clinical Outcome

Patients in both the intervention and control groups showed an improvement in quality of life (DLQI) at weeks 12 (V2) and 24 (V3; week 12 [V2]: *r*=–0.56; *P*=.006; week 24 [V3]: *r*=–0.86; *P*<.001; Figure 2; Table 2) compared with the baseline visits. No significant differences were observed between the control and intervention groups (*r*=–0.23; *P*=.42) and their progress (week 12 [V2]: *r*=0.45; *P*=.09; week 24 [V3]: *r*=0.42; *P*=.11; Table 2), although the intervention group showed a greater improvement than the control group.

Regarding pain, patients in both groups showed no significant amelioration over time compared with the baseline visits (V2: *r*=0.48; *P*=.48; V3: *r*=–0.74; *P*=.28; Figure 2; Table 2). There were no significant differences between the intervention and control groups (*r*=0.46; *P*=.53) and their trajectories (V2: *r*=0.11; *P*=.90; V3: *r*=0.96; *P*=.27; Table 2).

A significant improvement was observed in the activity score from V1 until V3 (V2: *r*=–1.39; *P*=.04; V3: *r*=–2.35; *P*=.001; Figure 2; Table 2). There was no difference between the 2 groups (*r*=0.08; *P*=.92) and their progress (V2: *r*=1.09; *P*=.21; V3: *r*=0.99; *P*=.26; Table 2).

There was also a significant improvement in the severity of eczema as assessed by the HECSI in both groups compared with the baseline visits (V2: *r*=–0.51; *P*=.02; V3: *r*=–0.72; *P*=.002; Figure 2; Table 2). There was no difference between the groups (*r*=–0.16; *P*=.56) or their trajectories (V2: *r*=0.33; *P*=.26; V3: *r*=0.43; *P*=.14; Table 2). All results were independent of sex, age, or disease duration (model 1; Table 2). Table 1 shows mean values of the scales, whereas Figure 2 shows predictive margins (delta method).

**Table 2:** Random effect regression models over 24 weeks. Model 0 unadjusted, and model 1 adjusted for age, sex, and disease duration (n=59; observations=177).

Assessment			Model 0					Model 1		
			*r* (SE)		*P* value		*r* (SE)			*P* value
**DLQI^a^**										
	Week 0	Ref^b^		Ref		Ref			Ref	
	Week 12	–0.561 (0.205)		.006		–0.561 (0.205)			.006	
	Week 24	–0.855 (0.205)		<.001		–0.855 (0.205)			<.001	
	Intervention group	Ref		Ref		Ref			Ref	
	Control group	–0.234 (0.288)		.42		–0.128 (0.288)			.66	
	Week 0 × control	Ref		Ref		Ref			Ref	
	Week 12 × control	0.450 (0.262)		.09		0.450 (0.262)			.09	
	Week 24 × control	0.420 (0.262)		.11		0.420 (0.262)			.11	
	*R*^2^: within	0.184 (N/A^c^)		N/A		0.184 (N/A)			N/A	
	*R*^2^: between	0.01 (N/A)		N/A		0.097 (N/A)			N/A	
	*R*^2^: overall	0.059 (N/A)		N/A		0.125 (N/A)			N/A	
**Pain**										
	Week 0	Ref		Ref		Ref			Ref	
	Week 12	0.478 (0.676)		.48		0.478 (0.676)			.48	
	Week 24	–0.739 (0.676)		.28		–0.739 (0.676)			.28	
	Intervention group	Ref		Ref		Ref			Ref	
	Control group	0.459 (0.723)		.53		0.468 (0.737)			.53	
	Week 0 × control group	Ref		Ref		Ref			Ref	
	Week 12 × control group	0.105 (0.866)		.90		0.105 (0.866)			.90	
	Week 24 × control group	0.961 (0.866)		.27		0.961 (0.866)			.27	
	*R*^2^: within	0.038 (N/A)		N/A		0.038 (N/A)			N/A	
	*R*^2^: between	0.040 (N/A)		N/A		0.086 (N/A)			N/A	
	*R*^2^: overall	0.039 (N/A)		N/A		0.064 (N/A)			N/A	
**Activity**										
	Week 0	Ref		Ref		Ref			Ref	
	Week 12	–1.390 (0.677)		.04		–1.390 (0.677)			.04	
	Week 24	–2.350 (0.677)		.001		–2.350 (0.677)			<.001	
	Intervention group	Ref		Ref		Ref			Ref	
	Control group	0.079 (0.765)		.92		0.393 (0.755)			.60	
	Week 0 × control group	Ref		Ref		Ref			Ref	
	Week 12 × control group	1.090 (0.867)		.21		1.090 (0.867)			.21	
	Week 24 × control group	0.987 (0.867)		.26		0.987 (0.867)			.26	
	*R*^2^: within	0.144 (N/A)		N/A		0.144 (N/A)			N/A	
	*R*^2^: between	0.030 (N/A)		N/A		0.157 (N/A)			N/A	
	*R*^2^: overall	0.082 (N/A)		N/A		0.151 (N/A)			N/A	
**HECSI^d^**										
	Week 0	Ref		Ref		Ref			Ref	
	Week 12	–0.513 (0.229)		.03		–0.513 (0.229)			.03	
	Week 24	–0.715 (0.229)		.002		–0.715 (0.229)			.002	
	Intervention group	Ref		Ref		Ref			Ref	
	Control group	–0.158 (0.273)		.56		–0.062 (0.254)			.81	
	Week 0 × control group	Ref		Ref		Ref			Ref	
	Week 12 × control group	0.327 (0.293)		.26		0.327 (0.293)			.26	
	Week 24 × control group	0.429 (0.293)		.14		0.429 (0.293)			.14	
	*R*^2^: within	0.102 (N/A)		N/A		0.102 (N/A)			N/A	
	*R*^2^: between	0.01 (N/A)		N/A		0.286 (N/A)			N/A	
	*R*^2^: overall	0.044 (N/A)		N/A		0.211 (N/A)			N/A	

^a^DLQI: Dermatology Life Quality Index.

^b^Ref: reference value.

^c^N/A: not applicable.

^d^HECSI: Hand Eczema Severity Index.

### An App Use Frequency of Fewer Than Once Every 5 Weeks Leads to a Significant Amelioration of Quality of Life, Pain, Activity, and Extent of Eczema

When analyzing the outcomes in regard to app use frequency, the subgroup with an app use frequency of <20% showed a highly significant improvement in quality of life (DLQI) compared with the control group (V2: *r*=–1.23; *P*=.001; V3: *r*=–0.73; *P*=.05; Figure 3; Table 3). Overall, <20% app use means an app use frequency of <5 times over the study period. For the subgroup with ≥20% app use, there was no significant difference in the DLQI score compared with the control group (V2: *r*=–0.03; *P*=.91; V3: *r*=0.25; *P*=.39; Figure 3; Table 3).

The pain also improved significantly in the subgroup with <20% app use frequency compared with the control group (V2: *r*=–2.96; *P*=.02; V3: *r*=–2.97; *P*=.02; Figure 3; Table 3). In the subgroup with ≥20% app use frequency, there was again no significant effect (V2: *r*=1.41; *P*=.14; V3: *r*=–0.11; *P*=.91; Figure 3; Table 3).

In regard to the activity score of the patients, a significant improvement in the subgroup with <20% app use frequency in comparison with the control group was noted for V2, but not for V3 (V2: *r*=–3.07; *P*=.01; V3: *r*=–1.76; *P*=.17; Figure 3; Table 3). There were no significant differences in the subgroup with ≥20% app use frequency (V2: *r*=–0.03; *P*=.98; V3: *r*=–0.57; *P*=.56; Figure 3; Table 3).

The HECSI showed a significant improvement in the subgroup with <20% app use frequency in comparison with the control group at V2 but again not at V3 (V2: *r*=–0.99; *P*=.02; V3: *r*=–0.65; *P*=.12; Figure 3; Table 3). There were again no significant differences in the subgroup with ≥20% app use frequency in comparison with the control group (V2: *r*=0.03; *P*=.94; V3: *r*=–0.31; *P*=.35; Figure 3; Table 3). Again, all results were independent of sex, age, or disease duration (model 1; Table 3).

**Table 3:** Random effect regression models of the app use frequency subgroups <20% and ≥20% over 24 weeks. Model 0 unadjusted, and model 1 adjusted for age, sex, and disease duration (n=59; observations=177).

Assessment		Model 0			Model 1	
		*r* (SE)	*P* value	*r* (SE)		*P* value
**DLQI^a^**						
	Week 0	Ref^b^	Ref	Ref		Ref
	Week 12	–0.110 (0.159)	.49	–0.110 (0.159)		.49
	Week 24	–0.435 (0.159)	.006	–0.440 (0.159)		.006
	Control group	Ref	Ref	Ref		Ref
	Intervention group with <20% app use frequency	0.524 (0.421)	.21	0.531 (0.419)		.21
	Intervention group with ≥20% app use frequency	0.079 (0.331)	.81	–0.089 (0.337)		.79
	Week 0 × control group	Ref	Ref	Ref		Ref
	Week 12 × intervention group with <20% app use frequency	–1.230 (0.373)	.001	–1.230 (0.373)		.001
	Week 12 × intervention group with ≥20% app use frequency	–0.032 (0.293)	.91	–0.032 (0.292)		.91
	Week 24 × intervention group with <20% app use frequency	–0.733 (0.373)	.049	–0.733 (0.373)		.049
	Week 24 × intervention group with ≥20% app use frequency	–0.253 (0.293)	.39	–0.253 (0.292)		.39
**Pain**						
	Week 0	Ref	Ref	Ref		Ref
	Week 12	0.583 (0.521)	.26	0.583 (0.521)		.26
	Week 24	0.222 (0.521)	.67	0.222 (0.521)		.67
	Control group	Ref	Ref	Ref		Ref
	Intervention group with <20% app use frequency	1.390 (1.050)	.19	1.600 (1.060)		.13
	Intervention group with ≥20% app use frequency	–1.440 (0.827)	.08	–1.590 (0.846)		.06
	Week 0 × control group	Ref	Ref	Ref		Ref
	Week 12 × intervention group with <20% app use frequency	–2.960 (1.220)	.02	–2.960 (1.220)		.02
	Week 12 × intervention group with ≥20% app use frequency	1.420 (0.961)	.14	1.420 (0.961)		.14
	Week 24 × intervention group with <20% app use frequency	–2.970 (1.220)	.02	–2.970 (1.220)		.02
	Week 24 × intervention group with ≥20% app use frequency	0.111 (0.961)	.91	0.111 (0.961)		.91
**Activity**						
	Week 0	Ref	Ref	Ref		Ref
	Week 12	–0.306 (0.535)	.57	–0.306 (0.535)		.57
	Week 24	–1.360 (0.534)	.01	–1.360 (0.535)		.01
	Control group	Ref	Ref	Ref		Ref
	Intervention group with <20% app use frequency	0.764 (1.120)	.50	0.792 (1.100)		.47
	Intervention group with ≥20% app use frequency	–0.572 (0.880)	.55	–1.04 (0.880)		.24
	Week 0 × control group	Ref	Ref	Ref		Ref
	Week 12 × intervention group with <20% app use frequency	–3.070 (1.250)	.01	–3.070 (1.250)		.01
	Week 12 × intervention group with ≥20% app use frequency	–0.028 (0.986)	.98	–0.028 (0.986)		.98
	Week 24 × intervention group with <20% app use frequency	–1.760 (1.250)	.17	–1.760 (1.250)		.16
	Week 24 × intervention group with ≥20% app use frequency	–0.572 (0.986)	.56	–0.572 (0.986)		.56
**HECSI^c^**						
	Week 0	Ref	Ref	Ref		Ref
	Week 12	–0.185 (0.181)	.31	–0.185 (0.181)		.31
	Week 24	–0.286 (0.181)	.11	–0.286 (0.181)		.11
	Control group	Ref	Ref	Ref		Ref
	Intervention group with <20% app use frequency	0.383 (0.399)	.34	0.466 (0.371)		.21
	Intervention group with ≥20% app use frequency	0.037 (0.314)	.91	–0.160 (0.296)		.59
	Week 0 × control group	Ref	Ref	Ref		Ref
	Week 12 × intervention group with <20% app use frequency	–0.990 (0.423)	.02	–0.990 (0.423)		.02
	Week 12 × intervention group with ≥20% app use frequency	0.026 (0.333)	.94	0.026 (0.333)		.94
	Week 24 × intervention group with <20% app use frequency	–0.652 (0.423)	.12	–0.652 (0.423)		.12
	Week 24 × intervention group with ≥20% app use frequency	–0.310 (0.333)	.35	–0.310 (0.333)		.35

^a^DLQI: Dermatology Life Quality Index.

^b^Ref: reference value.

^c^HECSI: Hand Eczema Severity Index.

### Male Patients Profit More From the Intervention Regarding the Clinical Outcome

In a further subgroup analysis of the intervention group in regard to the sex-specific development of the HECSI, we found a significant improvement in the HECSI compared with baseline only for male participants (V2: *r*=–1.06; *P*=.008; V3: *r*=–1.21; *P*=.003).

### Correlation of the eHECSI With the HECSI

Correlating the eHECSI assessed on the basis of pictures taken by the patients of their hands and feet with the HECSI recorded by physicians during regular personal visits, the eHECSI correlated strongly with the in-person–assessed HECSI (*r*=0.898; *P*=.002) even when the quality of the images was not that good. If the pictures were of good quality, the correlation of the eHECSI with the HECSI was also highly significant (*r*=0.875; *P*<.001).

## Discussion

### Principal Findings

In our intervention study, we showed that the use of our monitoring app in combination with a patient education session has a significant effect on quality of life, pain, activity, and clinical outcome if the app is not used more than once every 5 weeks. In addition, men seem to profit more from app use frequency than women regarding the clinical outcome.

We first analyzed differences between the intervention and control groups in regard to amelioration of quality of life, pain, activity, and eczema. All our study patients, independent of group membership, had less pain, showed an enhanced quality of life, and participated more actively in life; in addition, their skin condition improved over time. Although the intervention group showed a stronger improvement at all times, the difference between the 2 groups never reached significance. As our patients received a physician’s appointment every 3 months regardless of their skin condition, we conclude that the regular physician-patient contact was crucial for the amelioration of the disease in both groups. This aligns with the observations of Riedl et al [18] who showed that regular physician-patient contact leads to improvement in subjective and objective symptoms. Direct physician-patient contact seems to be more effective than an educational program combined with a monitoring app in the short term regarding our whole study population. In this case, the final evaluation of the study data at week 60 will provide better knowledge about the long-term effects achieved by our intervention.

In our previous intervention study involving a 60-week monitoring app for patients with psoriasis, we were able to show that patient education in combination with a monitoring app resulted in a significant amelioration of depressive and anxiety symptoms in patients who used the app fewer than once a month [17]. In that study, we concluded that patients who were chronically ill do not wish to be reminded of their disease too often. Moreover, it seemed that patients do not want to invest too much time in documenting their disease because they already need to spend considerable time in taking care of their eczematous skin. Furthermore, in this study, an app use frequency of fewer than once every 5 weeks led to a significant amelioration of quality of life, pain, activity, and extent of eczema in the subgroup using the app fewer than once a month (<20% app use frequency) compared with the control group. The mainstay of hand and foot eczema management is still topical therapy, which needs to be applied several times a day. For patients with psoriasis, process aspects such as application time have been associated with nonadherence and a negative impact on quality of life [19,20]. In line with this observation, Retzler et al [21] showed that topical treatment regimens in patients with atopic dermatitis have a detrimental effect on quality of life that increases with treatment duration and frequency of application. Therefore, an additional time-consuming burden imposed on patients with hand and foot eczema such as a too-frequent app-based documentation of their skin disease might generate no additional benefits regarding quality of life and disease outcome. It should be noted that the collected data do not allow differentiating whether patients who used the app less frequently simply experienced an improvement in their skin condition. This group could have profited solely from the patient education, which enhanced knowledge, provided in the study. This observation is in concordance with the study by Ahn et al [22], who were able to show that patient education and web-based resources in dermatology increase compliance and adherence to therapy. We cannot rule out that the education provided by the 2 dermatological specialists led to the assessed significant improvement in the subgroup using the app fewer than once every 5 weeks, but in our previous study [12] for patients with psoriasis, the education alone had no effect on the outcome. Therefore, we assume that the same is true for patients with chronic hand and foot eczema.

We additionally assessed whether patients reduce the app use frequency as their outcomes improve, but the subgroup with <20% app use frequency showed lower app use frequency from the start, with no decrease in the use in the course of time.

By contrast, the app provided in the study allowed direct contact between patients and their treating physicians, which probably reassured patients and improved quality of life in the intervention group when using the app fewer than once a week. We believe that the mere possibility of being able to contact the supervising physician if needed rather than the frequency of physician-patient contact is decisive to improved quality of life. In our clinical perception, frequent physician’s appointments to obtain a follow-up prescription may become a burden, in particular for younger patients who have less time because of their jobs. Such patients might benefit significantly from additional teledermatological care.

Another finding of our study was that the HECSI of male participants decreased faster than those of female participants, independent of app use frequency, although women show higher adherence to topical therapy [23]. We assume that men may benefit more from a constant reminder to apply their topical therapy provided by an eHealth device even when they avoid frequent documentation of their eczema in the app. A positive benefit for reminder apps has already been demonstrated for therapy adherence in patients with cardiovascular disease [24]. Further studies addressing this point are needed in patients with hand and foot eczema.

One of the study’s great strengths was that we were able to show that telemedical care can at least partially replace personal care in patients with hand and foot eczema because the analysis of pictures taken by the patients correlates strongly with that of the in vivo images. Therefore, the HECSI assessed in the face-to-face visit correlated significantly with the eHECSI. This is surely not the case for all dermatological diseases in which the disease can affect the whole body, especially the genital area and the capillitium. A study by Zabludovska et al [25] concluded that only significant changes were detected by photographs; however, in the study, the number of participants was very small (N=33). Whether photographs can be used to monitor the progression of chronic hand eczema and reliably determine HECSI should be further investigated.

Our study includes some limitations. A major limitation is the monocentric design and the small study cohort, which limits generalizability of the results. In particular, the group with <20% app use frequency is very small, which could have led to missed or overinterpreted differences between the groups, especially as we compared this subgroup of the intervention group with the control group. Further studies are necessary to verify our findings on a broader scale.

### Conclusions

Overall, our intervention had a positive effect on quality of life, pain, activity, and possibly the clinical outcome in a subgroup of patients with hand and foot eczema.

We were able to show that a monitoring app for patients with hand and foot eczema that allows direct contact with their treating physicians combined with patient education may have the potential to improve the eczema outcome of these patients, especially if the app is not used too frequently. We believe that a monitoring app such as the one presented in this study has the potential to improve patient care and should be implemented in daily practice. However, because of the small number of participants, especially in the subgroups of the intervention group, as well as missing data on treatment adherence of the control group, these data need to be re-examined in a larger sample with consideration of individual factors.

## Appendix

Multimedia Appendix 1 CONSORT-eHEALTH checklist (V 1.6.1).

## Abbreviations

DiGA: German acronym for Digital Health Applications
DLQI: Dermatology Life Quality Index
eHECSI: electronic Hand Eczema Severity Index
HECSI: Hand Eczema Severity Index
NRS: numeric rating scale
V: visit time point
